# Effects of Decision Aids on Decision Knowledge, Conflict, and Satisfaction Among Patients With Cancer: A Systematic Review and Meta‐Analysis

**DOI:** 10.1155/jonm/6436400

**Published:** 2026-05-14

**Authors:** Yang Chen, Chuanmei Zhu, Linna Li, Juejin Li, Qianwen Yan, Xiaolin Hu

**Affiliations:** ^1^ West China School of Nursing, West China Hospital, Sichuan University, Chengdu, Sichuan, China, scu.edu.cn; ^2^ Outpatient Department, West China Hospital, Sichuan University, Chengdu, Sichuan, China, scu.edu.cn; ^3^ Yong Loo Lin School of Medicine, Alice Lee Centre for Nursing Studies, National University of Singapore, Singapore, nus.edu.sg, nus.edu.sg; ^4^ Tianfu Jincheng Laboratory, City of Future Medicine, Chengdu, Sichuan, China, scu.edu.cn

## Abstract

**Aim:**

To examine the effects of decision aids (DAs) on decision knowledge, conflict, and satisfaction among patients with cancer.

**Background:**

DAs are frequently used to improve the quality of health decision‐making across various cancer types (e.g., breast, prostate, and colorectal cancer). However, the impact of DAs on decision knowledge, conflict, and satisfaction remains unclear, and the various delivery methods (e.g., telephone, brochure, mixed delivery methods, and web‐based delivery methods) exhibit inconsistent effects.

**Methods:**

We systematically searched eight databases and two search engines from inception to November 30, 2025, for randomized controlled trials (RCTs), evaluating DAs in adult cancer patients facing treatment decisions. A frequentist random‐effects meta‐analysis was conducted using Stata 16.0 to synthesize the primary outcomes of decision knowledge, conflict, and satisfaction. Pooled effects were estimated as standardized mean differences (SMDs) with 95% confidence intervals (CIs). Heterogeneity was explored via subgroup and sensitivity analyses. Publication bias was assessed using funnel plots and Egger’s test, with the trim‐and‐fill method applied when indicated. The methodological quality of individual studies was assessed using the Cochrane Risk‐of‐Bias Tool 2.0 (ROB 2), and the overall certainty of the evidence for each outcome was evaluated using the Grading of Recommendations Assessment, Development and Evaluation (GRADE) framework.

**Results:**

Thirty RCTs with 4303 cancer patients were included. The pooled analysis showed a positive effect on decision knowledge (SMD = 0.91, 95% CI: 0.49–1.33) and a reduction in decision conflict (SMD = −0.23, 95% CI: −0.39 to −0.07). No significant effect was observed on decision satisfaction (SMD = 0.03, 95% CI: −0.42–0.48). In subgroup analyses, all three delivery methods (brochure, web‐based, and mixed) were associated with increased decision knowledge, while only web‐based and mixed methods were associated with reduced decision conflict. DAs designed for both specific and various cancer types were associated with improved knowledge, whereas only those targeting a specific cancer type were associated with reduced conflict.

**Conclusion:**

DAs are effective in improving decision knowledge and reducing decision conflict, but they do not effectively increase decision satisfaction.

**Implications for Nursing Management:**

Nurses should integrate evidence‐based DAs, particularly web‐based and mixed delivery formats, into routine cancer care to enhance patients’ decision knowledge and reduce conflict. To maximize effectiveness, DAs should be tailored based on targeted cancer types, educational levels, and health literacy to achieve unmet needs in decision satisfaction.

## 1. Introduction

Due to advancements in diagnostic technologies and therapeutic options, the number of detected cancer cases has increased [1, 2]. Cancer treatment decisions are often particularly challenging for patients to comprehend and navigate, owing to the complexity of clinical information (e.g., disease stage, tumor size, and location) and heterogeneous patient profiles (e.g., educational level, health literacy, socioeconomic status, values, and preferences). Moreover, cancer care frequently involves high‐stakes trade‐offs between survival and quality of life, rapidly evolving treatment options and emotionally charged decision contexts—all of which can overwhelm patients and complicate shared decision‐making (SDM) [3]. For nurses, it is worth considering how to effectively convey information about diseases to their patients within the limited time [4]. Decision knowledge is one of the crucial factors influencing patients to make decisions [5]. The primary method for cancer patients to acquire decision knowledge is to consult with their health providers. However, patients face barriers when raising a question, such as fear of negative comments, confusion about medical terminology, and perceptions that the nurses and doctors are “too busy” [6]. Moreover, decision conflict—conceptualized as a state of uncertainty about which course of action to choose when options involve risks, losses, or challenges to personal values—is a common experience among patients facing preference‐sensitive decisions [7]. It typically arises from factors such as a lack of clear information about available options and their potential benefits and harms, uncertainty about one’s own values and preferences, and perceived insufficient support or guidance in the decision‐making process [8, 9]. In addition to the former two outcome indicators, decision satisfaction is also an important consideration that reflects the decision quality and patients’ decision‐making experience [10, 11]. Prior researchers have shown that cancer patients express dissatisfaction with the decisions they make and the results they achieve when relying solely on their own capabilities [12]. They felt that they did not acquire sufficient knowledge at that time, were not clear about their own values, and had unaddressed concerns [13]. When cancer patients have inadequate decision knowledge, they will experience negative emotions, which will influence treatment adherence [14]. Therefore, it is paramount to conduct interventions to help cancer patients in the process of decision‐making and fully reflect their individual preferences, thus facilitating subsequent treatment and rehabilitation.

SDM is a collaborative process in which clinical healthcare personnel and patients jointly participate in medical decisions based on patients’ values and preferences and ultimately come to a mutual agreement [15]. Decision Aids (DAs) play a crucial part in enabling SDM and enhancing decision quality for preference‐sensitive decisions [16]. They assist individuals in the process of SDM by offering information on the available choices and potential results, clearly outlining the decisions that need to be made and helping to clarify personal values and preferences [17]. While formal evidence‐based guidelines could provide professional medical knowledge and optimal clinical practice, individual profiles also need to be taken into account [18]. Common delivery methods of DAs include brochures (pamphlets/leaflets), websites, and mixed delivery methods (such as the combination of an instructional video followed by a booklet or a brochure accompanied by a structured PowerPoint presentation) [19]. By providing structured information and value‐clarification exercises, DAs help patients understand the options and the personal importance of potential outcomes, thereby preparing them to actively participate in SDM with their clinicians. These DAs greatly promote decision quality and lead to better patient–clinician communication [20].

Due to these advantages, SDM, aided by DAs, has been widely used in various clinical fields. These include reducing the bias of patients and families toward hospice care, clarifying treatment preference and values in type 2 diabetes, and improving readiness to transition to alternative therapies for patients with rheumatoid arthritis who fail on their current treatment [21–23]. For patients with cancer, there has been a noteworthy increase in the utilization of DAs in oncology practice to support patients in making decisions. For example, helping newly diagnosed breast cancer patients considering whether to undergo mastectomy, supporting young female cancer patients in decision‐making regarding future fertility, and providing with resources to help facilitate patients’ ability to make an informed decision about whether or not to participate cancer clinical trials [24–26]. However, while DAs generally improve decision knowledge and reduce conflict, their effects on satisfaction are inconsistent. Liao et al. demonstrated that DAs had no significant effect on decision satisfaction among patients with hepatocellular cancer, whereas Whelan et al. indicated that breast cancer patients in the DA’s group reported higher satisfaction with decision‐making [27, 28]. Existing meta‐analysis on DAs predominantly focused on single cancer type (e.g., breast or prostate cancer), with a limited number of studies exploring the influence on the overall cancer populations [29, 30]. Therefore, this systematic review and meta‐analysis aim to synthesize the existing evidence to evaluate the overall effects of DAs on decision‐related outcomes—knowledge, conflict, and satisfaction among patients with cancer. Moreover, we employed subgroup analysis to explore heterogeneity between delivery methods, conducted region, and whether designed specifically for targeted cancer population.

## 2. Method

PROSPERO has received registration for this systematic review and meta‐analysis (CRD42024519828). The findings were reported in step with the revised Preferred Reporting Items for Systematic Review and Meta‐Analysis (PRISMA) guidelines [31].

### 2.1. Data Sources and Search Strategies

We comprehensively searched eight databases and two search engines from inception to November 30, 2025. The four English databases include Web of Science Core Collection, Cochrane Central Register of Controlled Trials (CENTRAL), PsycINFO, and Cumulative Index to Nursing and Allied Health Literature (CINAHL), while the four Chinese databases are China Biology Medicine disc (CBMdisc), China National Knowledge Infrastructure (CNKI), VIP Database for Chinese Technical Periodicals (VIP), and WanFang Data (WanFang). The two search engines utilized are PubMed and Embase. The search strategies were developed in accordance with the Cochrane methodology and included Medical Subject Heading (MeSH) items, text words, and Boolean operators. Searches combined terms related to (1) neoplasms (e.g., “cancer,” “tumor,” “carcinoma,” “oncology”) and (2) DA (e.g., “decision support,” “shared decision‐making”). Supporting Table S1 contains the complete search strategies for each database and search engine. Only RCTs and human subjects were involved.

### 2.2. Criteria for Eligibility and Exclusion

The eligibility criteria of this review were performed in line with the Participants, Intervention, Comparison, Outcome and Study design (PICOS) structure as follows:

 Participants (P): adult patients (aged 18 years or older) who were diagnosed with cancer and were facing cancer‐related clinical decisions.
 Intervention (I): experimental groups using DAs, which we define as tools that present information on cancer‐related options and the specific outcomes of each choice, which can assist cancer patients to make decisions based on their own values and preferences.
 Control (C): the control group was treated with usual care (standard care or therapies that did not offer DAs), including attention control conditions or wait‐list.
 Outcomes (O): studies were eligible if they measured at least one of the following outcomes at any time point following the decision‐making encounter, operationally defined as
   Decision knowledge: the objective understanding of disease and treatment information, measured by the proportion of correct answers on knowledge questionnaire.
   Decision conflict: the level of uncertainty about a course of action, measured by the Decision Conflict Scale (DCS).
   Decision satisfaction: the contentment with the decision or decision process, measured by scales such as the Satisfaction with Decision (SWD).
 Study design [S]: clinical RCTs published in English language. We excluded studies that met any of the following criteria: inaccessible full text, protocols, reviews, conference abstracts, quasi‐experimental studies, case studies, or qualitative studies.

### 2.3. Selection of Studies and Extraction of Data

Two reviewers (YC and QWY) first independently retrieved and screened the studies using EndNote 20 (Clarivate Analytics). They then independently extracted the data from the included studies. After the exclusion of duplicate articles, the remaining articles were assessed based on their titles and abstracts.

Subsequently, potentially qualified studies were subjected to full‐text screening to determine whether they satisfied the eligibility criteria. Then, reviewers extracted the following information from the included articles: study description (the first author, year, country); participant profile (mean age, cancer types, sample size, gender); intervention and control group particulars (intervention introductions, delivery methods, duration); and outcome information (results, measurements, assessment time). Eventually, all the data were collected in an Excel worksheet. Any dispute over the inclusion of a study or data extraction was discussed with the third author.

### 2.4. Data Synthesis and Analysis

Statistical analyses were performed using Stata 16.0 software. For continuous outcomes, the mean, standard deviation (SD), and sample size were extracted from each study to calculate the effect size. The standardized mean difference (SMD) was used to pool continuous data across studies, presented alongside their 95% confidence intervals (CIs), which estimate the range within which the true effect size is likely to lie [32, 33]. A random‐effects model was employed as the primary analytical framework for meta‐analyses, using the DerSimonian–Laird (DL) estimator to account for anticipated heterogeneity arising from differences in study populations and interventions [34]. A random‐effects model was employed for all meta‐analysis, as it incorporates the assumption that the true effect may vary across studies due to methodological or clinical diversity. Heterogeneity among the included studies was quantified using the *I* ^2^ statistic. *I* ^2^ values of 25%, 50%, and 75% were interpreted as representing low, moderate, and high heterogeneity, respectively. While the random‐effects model was used irrespective of the *I* ^2^ value, the degree of heterogeneity was reported and considered in the interpretation of results. For multiarm trials that shared a common control group, the control group was split into two subgroups of approximately equal size to create independent pairwise comparisons for the meta‐analysis. This approach prevents the double‐counting of participants in the control group and ensure the independence of effect sizes [35].

Subgroup analyses were prespecified to explore potential sources of heterogeneity based on clinical and methodological rationale. We specifically examined (1) the effectiveness of different delivery methods (brochure, web‐based, mixed methods), hypothesizing that interactive and multimodal formats might lead to greater engagement and knowledge retention compared to static materials; (2) the effect of cancer types (individual or various types), as the decision‐making context and treatment complexity vary substantially across cancers, which could influence the intervention’s impact; and (3) the effect of the region where DAs were conducted, considering that cultural and healthcare system factors might modify their acceptability and effectiveness. These analyses were considered exploratory, and all interpretations were made with caution due to the limited statistical power within subgroups and the increased risk of type I error from multiple comparisons. It is important to note that these subgroup analyses are exploratory in nature. To determine if the effect sizes differ significantly between subgroups, we will perform a formal test for interaction. A *p* value for interaction of < 0.05 will be considered statistically significant. The findings from these analyses will be interpreted with caution as they are observational in nature and may be confounded by other study characteristics. Moreover, if sufficient studies were presented for the outcome (*n* > 10), a funnel plot was generated to discern publication bias. For Egger’s test, a statistically significant *p* value < 0.05 was considered indicative of potential publication bias and the Duval and Tweedie’s trim‐and‐fill method would be employed to estimate the effect after accounting for potentially missing studies [36, 37]. The reliability of the results was assessed with sensitivity analysis by excluding individual studies from each forest plot.

### 2.5. Quality Assessment

We applied the Cochrane Risk of Bias tool 2 (RoB 2.0) at the study level for the outcomes synthesized in our meta‐analysis [38]. The “effect to interest” was defined based on the primary analysis presented in each study. For studies reporting an intention‐to‐treat (ITT) analysis, we assessed the “effect of assignment to the intervention.” For studies reporting a per‐protocol (PP), we assessed the “effect of adhering to the intervention.” This distinction guided the assessment of Domain 2 (deviations from intended interventions) accordingly. The revised tool comprises five domains, namely, (1) randomization process, (2) deviations from intended interventions, (3) missing outcome data, (4) measurement of the outcome, and (5) selection of the reporting results. In each respective domain, several questions need to be answered using five replies: “Yes (Y),” “Probably Yes (PY),” “Probably No (PN),” “No (N),” and “No information (NI).” Each domain was evaluated as “low risk of bias”, “some concerns of bias,” or “high risk of bias.” In the overall evaluation, a study was considered to have a “low risk of bias” if all fields signified a low risk of bias. A study was deemed to have “some concerns of bias” if there were concerns in at least one field without a high risk of bias in any field. A study was classified as having a “high risk of bias” if there was a high risk of bias in at least one field or if there were concerns in multiple fields. The assessment was conducted independently for each outcome. The quality of the studies was assessed by two reviewers separately, and any discrepancies were reconciled with a third author. The certainty of evidence for outcomes related to decision knowledge, decision conflict, and decision satisfaction was evaluated using the Grading of Recommendations Assessment, Development and Evaluation (GRADE) framework. Judgments were made based on the risk of bias, inconsistency, indirectness, imprecision, and publication bias. For imprecision, a minimal important difference (MID) was defined a priori: for continuous outcomes measured on a 0–100 scale, an MID of 10 points was considered clinically meaningful. Evidence was downgraded when the 95% confidence interval crossed this MID threshold. The overall certainty of evidence for each outcome is presented in the summary of findings table [32, 39].

## 3. Results

### 3.1. Selection of Studies

**FIGURE 1:**
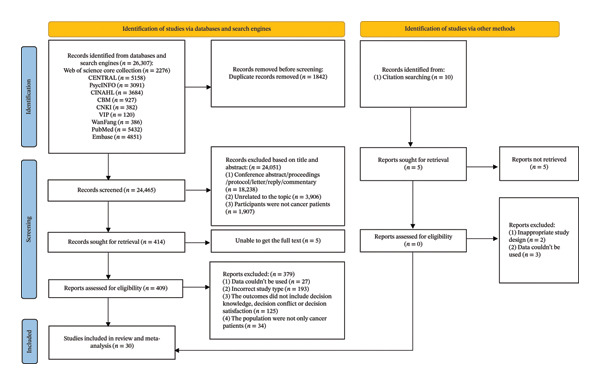
PRISMA 2020.

From an initial search yielding 26,307 publications, a total of 30 qualifying RCTs met the eligibility criteria and were included, comprising 4303 participants [24, 26–28, 37, 40–64]. The detailed study selection process is presented in Figure 1.

### 3.2. Characteristics of Studies

A total of 30 RCTs, involving 4303 participants with the sample sizes ranging from 20 [49] to 388 [53], were included in this review. The key characteristics of these studies are summarized in Table 1 (the complete detailed table is available in Supporting Table S2). They were posted between 2001 [44] and 2025 [64]. In terms of the conducted region, 21 studies were conducted in Western countries, including the United States (*n* = 9), Canada (*n* = 4), the UK (*n* = 2), the Netherlands (*n* = 2), Spain (*n* = 1), Switzerland (*n* = 1), Australia (*n* = 1), and German (*n* = 1). Nine studies were conducted in Asia, including China (*n* = 4), Singapore (*n* = 1), Malaysia (*n* = 1), India (*n* = 1), South Korea (*n* = 1), and Japan (*n* = 1). Twenty‐nine studies used a two‐arm RCT design, and only one study involved three groups: one received “standard care,” another received “standard care plus the Navya‐PPT followed by a research questionnaire completed by patients alone,” and a third group received “standard care plus the Navya‐PPT followed by a research questionnaire completed with a key family member present” [48]. The mean or median age of all cancer survivors was between 28.78 and 70.27 years [45, 47]. Of the 30 included studies, seven included individuals diagnosed with multiple types of cancer. The remaining 23 studies focused on individual cancer types, including breast cancer (*n* = 14), prostate cancer (*n* = 4), colorectal cancer (*n* = 2), hepatocellular cancer (*n* = 1), papillary thyroid cancer (*n* = 1), and lung cancer (*n* = 1).

**TABLE 1:** The main characteristics of the studies included in the meta‐analysis.

**First author, year, country**	**Participants**				
	Age, mean (SD)/mean (range), years**^a^**	**Type of cancer**	**Sample size**		**Delivery method**
			**E/C2**	**Male/female**	
Belkora et al., 2012 USA [37]	59.0 (12) 59.0 (9)	Breast	35/32	0/67	Telephone
Chabrera et al., 2015 Spain [40]	69.2 (5.99) 69.0 (5.83)	Prostate	61/61	122/0	Brochure
Chong et al., 2021 Singapore [41]	55 (29–83) 58.4 (22–75)	Cancer	120/120^a^	77/163	Brochure
Christy et al., 2022 USA [26]	54.3 (15.8) 54.0 (11.8)	Cancer	41/51	31/60	Web‐based
Cuypers et al., 2018 Netherlands [42]	64.9 (6.0) 66.3 (5.7)	Prostate	235/101^a^	336/0	Web‐based
Fan et al., 2019 China [43]	NA	Oral and Maxillofacial	60/60	72/48	Mixed (Brochure + PPT)
Goel et al., 2001 USA [44]	57.4 (12.75) 57.59 (11.96)	Breast	78/45	0/123	Mixed (Workbook + Audiotape)
Ehrbar et al., 2019 Switzerland [45]	29.92 (4.35) 28.78 (4.77)	Cancer	17/20	0/37	Web‐based
Hacking et al., 2013 UK [46]	65.4 67.2	Prostate	62/53^a^	115/0	Mixed (Navigator + CD)
Jalil et al., 2022 Malaysia [47]	68.96 (6.12) 70.27 (7.67)	Prostate	27/22^a^	49/0	Brochure
Joshi et al., 2023 India [48]	48 (28–70) 48 (28–72)	Breast	83/41^a^	0/124	Web‐based
Klifto et al., 2021 USA [49]	53.6 (7.9) 52.6 (10.6)	Breast	10/10	0/20	Brochure
Lam et al., 2014 China [50]	56.8 (10.8) 54.6 (10.1)	Breast	113/112	225/0	Brochure
Leighl et al., 2011 Canada [51]	61 62.5	Colorectal	107/100^a^	120/87	Brochure
Liao et al., 2023 China [27]	63.9 (11.3) 67.1 (13.5)	Hepatocellular carcinoma	35/34^a^	46/23	Mixed (Video + Leaflet)
Manne et al., 2019 USA [24]	47.5 (8.4) 45.5 (8.4)	Breast	39/44	0/83	Web‐based
Manne et al., 2015 USA [52]	51.7 (11.1) 48.2 (9.7)	Breast	21/22^a^	0/4	Web‐based
Marziliano et al., 2023 USA [53]	55.11 (10.64) 55.90 (11.60)	Breast	197/191	0/388	Mixed (Telephone + Internet + CD‐ROM)
Osaka et al., 2016 Japan [54]	49.7 (9.87) 48.6 (8.91)	Breast	61/55	0/116	Brochure
Politi et al., 2020 USA [55]	52.41 (9.99) 52.94 (9.35)	Cancer	92/88	74/132	Web‐based
Sawka et al., 2012 Canada [56]	47.5 (12.2) 44.1 (11.8)	Papillary thyroid	37/37^a^	12/62	Web‐based
Shepherd et al., 2019 UK [57]	62.71 (11.35) 61.5 (11.99)	Colorectal	65/67^a^	78/54	Mixed (Navigator + Audio recording + Compact disc)
Smith et al., 2020 USA [58]	53.6 (11)	Breast	110/142	0/252	Web‐based
Stege et al., 2023 Netherlands [59]	50.4 (11.0) 49.8 (11.1)	Breast	126/124	0/250	Web‐based
Stein et al., 2013 Australia [60]	66.3 (10.5) 66.3 (12.2)	Cancer	46/58	71/49	Brochure
Villalobos et al., 2024 German [61]	67 (46–88)	Lung	43/49	53/39	Brochure
Whelan et al., 2004 Canada [28]	58.2 58.1	Breast	94/107^a^	0/201	Mixed (Board + Cards)
Whelan et al., 2003 Canada [63]	51.0 51.8	Breast	82/93^a^	0/175	Mixed (Board + Cards)
Yun et al., 2019 Korea [63]	58.1 (11.9) 57.1 (11.0)	Cancer	104/100^a^	79/125	Mixed (Video + Booklet)
Wang et al., 2025 China [64]	43.3 (9.7) 46.2 (8.9)	Breast	32/31	0/63	Web‐based

*Note:* C: control group; E: experimental group; HCC: hepatocellular carcinoma; UC: usual care/standard care.

Abbreviations: AC, attention control; NR, not reported.

^a^At baseline.

#### 3.2.1. Characteristics of the DAs

The key characteristics of the 30 included DAs are summarized in Table 1, categorized by their primary delivery method. The table details the target population, core format, and critically, whether the aid reported outcome probabilities using absolute effect estimates and whether it communicated the certainty of the evidence (e.g., using GRADE or similar frameworks) to the user. Complete detailed characteristics of the included studies are shown in Supporting Table S2.

#### 3.2.2. Characteristics of the Control Groups

In four studies, participants in the control group were given attention control (an alternative intervention or contact of equal duration that did not contain the core elements of the DAs, such as browsing the National Cancer Institute’s CCT informational website) [26, 55, 63, 64], while in the other 26 studies, participants in the control group received the usual care throughout the study duration.

#### 3.2.3. Outcome Measurement Characteristics

The tools used to measure decision‐related outcomes varied considerably across studies in their specific focus.

##### 3.2.3.1. Decision Knowledge

The 13 distinct knowledge assessment tools primarily evaluated comprehension across three domains: (1) disease and treatment‐specific facts (e.g., understanding of hepatocellular carcinoma treatment options [27], breast cancer surgery procedures [48, 63]); (2) risk–benefit perceptions associated with various choices (e.g., probabilities of outcomes, side effects [24, 53]); and (3) system or procedural knowledge (e.g., health insurance concepts [56], advance care planning processes [64]). While most were study‐specific instruments, one employed a validated questionnaire [48]. One study did not report its measurement tool [50].

##### 3.2.3.2. Decision Conflict

The 16‐item DCS was the predominant measure, used in 22 studies. It comprehensively assesses dimensions such as uncertainty, informed choice, value clarity, support, and perceived decision quality. One study supplemented the DCS with a brief 4‐item screener [26]. The specific tool was unreported in one study [50].

##### 3.2.3.3. Decision Satisfaction

Satisfaction was measured using six different instruments focusing on related but distinct constructs: general satisfaction with the decision (Satisfaction With Decision Scale [27, 40, 51]), satisfaction with the information received (Satisfaction With Cancer Information Profile [42]), satisfaction with the decision‐making process itself (Participation Satisfaction in Medical Decision‐making Scale [43], effective decision‐making subscale [28, 52]), and satisfaction with clinical care [37].

### 3.3. Risk of Bias

The risk of bias was assessed for each of the three primary outcome domains (decision knowledge, decision conflict, and decision satisfaction) using the Cochrane RoB 2 tool. The overall judgments for each domain across all studies and outcomes are summarized in Figure 2, with detailed study‐by‐study assessments provided in Supporting Figure S1. Overall, the risk of bias was predominantly high or raised some concerns across all outcome domains. This pattern was largely driven by the domain of “Measurement of the outcome.” Given that the outcomes were self‐reported via questionnaires and participants/outcome assessors were typically not blinded to the intervention, most studies were judged as having a high risk of bias or some concerns in this domain for knowledge, conflict, and satisfaction outcomes. The domain of “Selection of the reported result” also raised frequent concerns. A total of 13 studies were judged to have some concerns in this domain, typically due to insufficient information in the trial protocol or registration record to confidently rule out selective reporting. In contrast, the domains of “Randomization process” and “Deviations from the intended interventions” were most frequently judged as low risk. Only a small number of studies were rated with some concerns (two and six studies, respectively) or high risk (four and one studies, respectively) in these areas.

FIGURE 2Risk‐of‐bias summary of the included studies. (a) Studies with the intention‐to‐treat analysis. (b) Studies with per‐protocol analysis.

**Figure:**
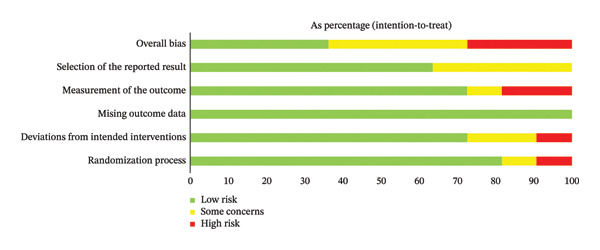
(a)

**Figure:**
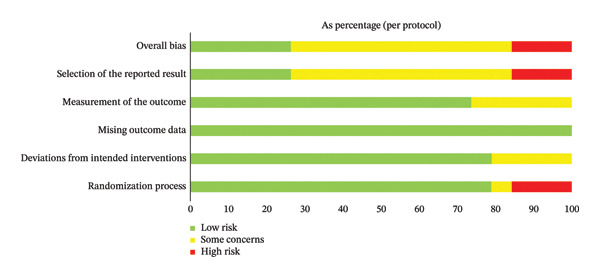
(b)

### 3.4. Quality of Evidence

Based on the GRADE framework, the evidence quality for decision knowledge was rated as low, whereas that for decision conflict and decision satisfaction was rated as moderate (Table 2).

**TABLE 2:** The GRADE assessment.

Outcomes	No. of studies	Risk of bias^a^	Inconsistency^b^	Indirectness^c^	Imprecision^d^	Other considerations	Overall certainty of evidence
Decision knowledge	15	Not Serious	Serious	Not serious	Not serious	None	⨁⨁◯◯ Low
Decision conflict	24	Not Serious	Serious	Not serious	Not serious	None	⨁⨁⨁◯ Moderate
Decision satisfaction	9	Not Serious	Serious	Not serious	Not serious	None	⨁⨁⨁◯ Moderate

^a^Certainty was downgraded if more than 50% of the weight of individual RCTs in each outcome assessed came from high‐risk studies.

^b^Based on the variability and heterogeneity across individual trials. Certainty was downgraded if *I* ^2^ > 50% and/or if the *p* value of the heterogeneity test was < 0.05.

^c^Assessed qualitatively by the extent to which the population, interventions, and outcome measures directly reflect the aims of the systematic review.

^d^Based on inspection of the pooled estimate and the 95% confidence interval (95% CIs). We decreased the grade rating by one (−1) when the analysis included fewer than 500.

### 3.5. Publication Bias

For decision conflict, Egger’s test did not show significant evidence of publication bias (*p* = 0.114). For decision knowledge, the results of Egger’s test suggested potential publication bias (*p* = 0.003). Given this evidence, we applied Duval and Tweedie’s trim‐and‐fill method to adjust for the potential effect of missing studies. The method imputed 15 theoretically missing studies to achieve a symmetrical funnel plot, with the adjusted pooled effect size changing from [0.49, 1.33] to [0.486, 1.327]. However, for decision satisfaction, the trim‐and‐fill method may be unreliable due to the number of studies being fewer than ten (*n* = 9). Although the trim‐and‐fill results did not alter the direction of the original pooled effects, suggesting that our findings are relatively robust, we cannot entirely rule out the possibility of publication bias given the limitation of the small sample size. More details are shown in Supporting Figures S2 and S3.

### 3.6. Meta‐Analysis

#### 3.6.1. Decision Knowledge

##### 3.6.1.1. Total Effects of DAs on Decision Knowledge

Fifteen studies (2121 participants) evaluated the effect of DAs on decision knowledge. The pooled analysis demonstrated a large improvement in knowledge favoring DAs over usual care (SMD = 0.91, 95% CI: 0.49–1.33) (Figure 3(a)). However, the evidence was highly heterogeneous (*I* ^2^ = 95%) and imprecise, as reflected in the wide confidence interval. The certainty of evidence for this outcome is reported in Table 2.

FIGURE 3Forest plot of total effects of decision aids on (a) decision knowledge, (b) decision conflict, and (c) decision satisfaction. CI = confidence interval, SD = standard deviation.

**Figure:**
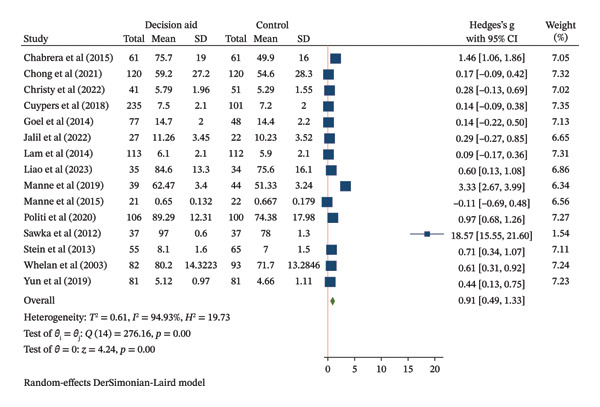
(a)

**Figure:**
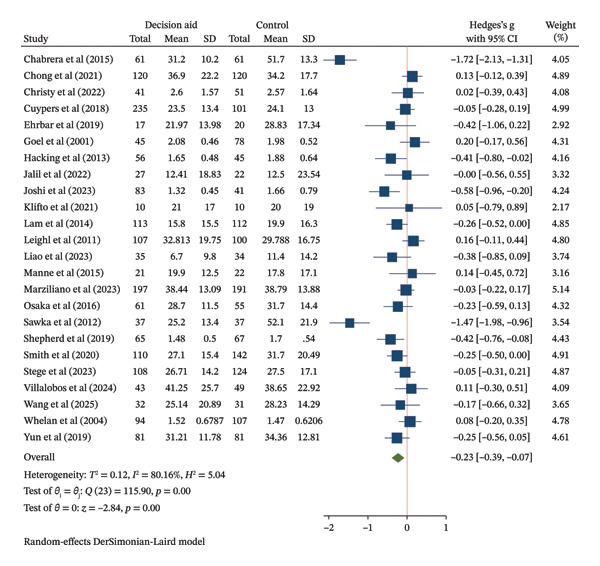
(b)

**Figure:**
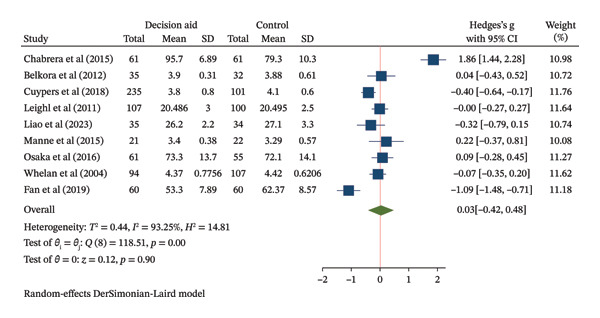
(c)

##### 3.6.1.2. Subgroup Analysis

In exploratory analyses by delivery method, the point estimate for knowledge improvement was highest for web‐based DAs (SMD = 2.43, 95% CI: 1.22–3.64), followed by mixed methods (SMD = 0.54, 95% CI: 0.34–0.74) and brochure‐based DAs (SMD = 0.47, 95% CI: 0.07–0.87) (Figure 4(a) (A)). However, the confidence intervals for these subgroups substantially overlapped, providing no clear evidence of a differential effect based on delivery method. A similar pattern of overlapping CIs was observed in the analysis by cancer‐type specificity, suggesting no evidence of a modifying effect (Figure 4(b) (A)).

FIGURE 4(a) Forest plots of the effects of the delivery methods of decision aids on (A) decision knowledge and (B) decision conflict. (b) Forest plot of the effects of whether they were specifically designed for a targeted cancer population of decision aids on (A) decision knowledge and (B) decision conflict. (c) Forest plot of the effects of conducted region of decision aids on decision conflict.

**Figure:**
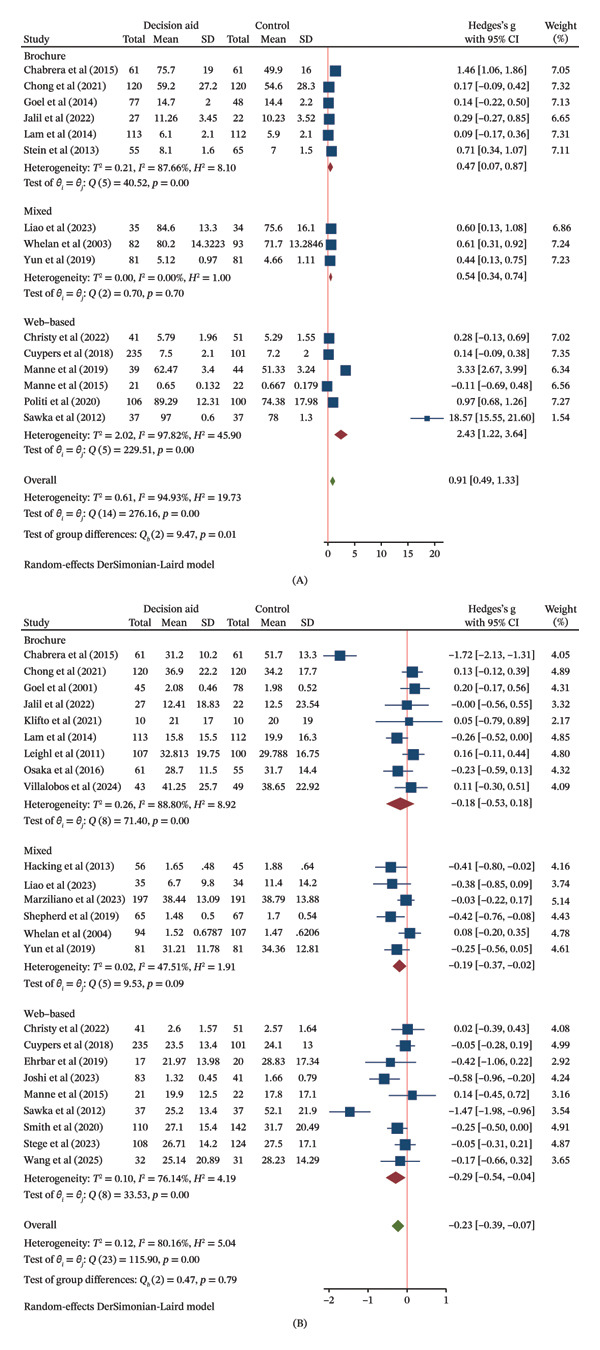
(a)

**Figure:**
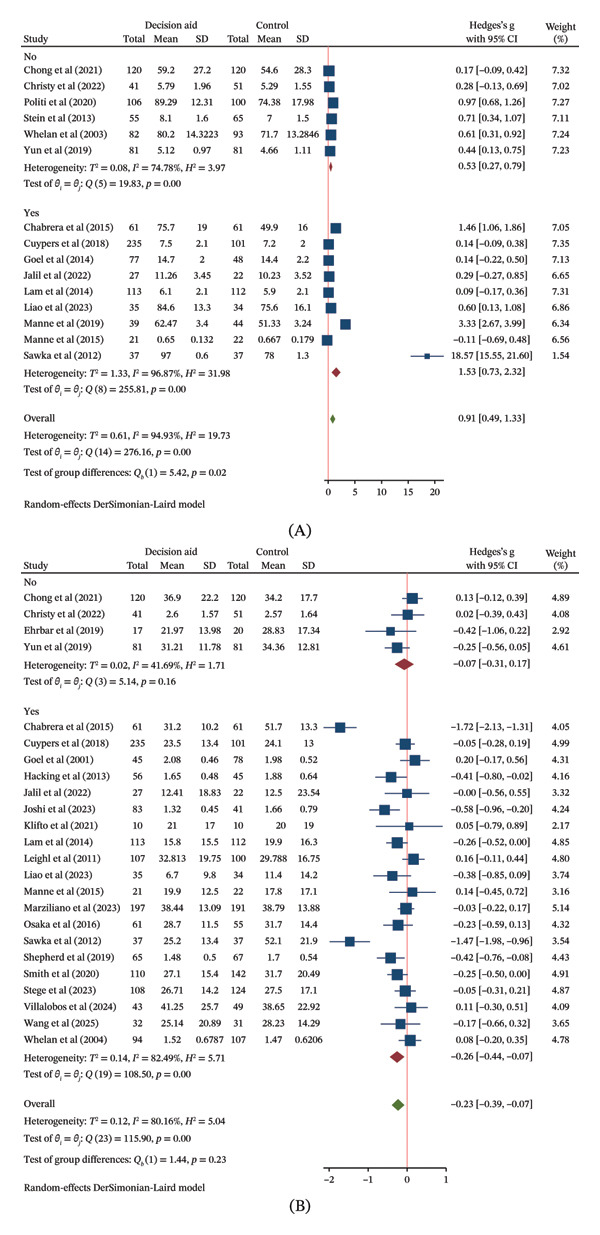
(b)

**Figure:**
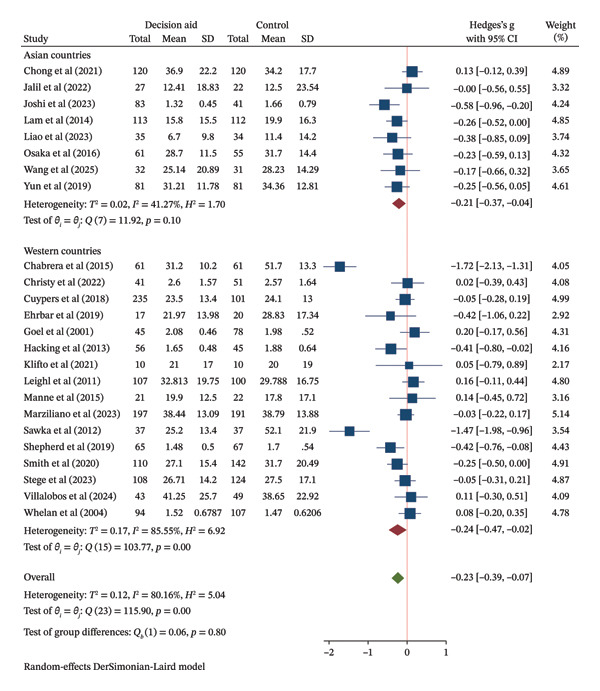
(c)

#### 3.6.2. Decision Conflict

##### 3.6.2.1. Total Effects of DAs on Decision Conflict

Twenty‐four studies reported on decision conflict. The pooled analysis showed that DAs reduced decision conflict compared to usual care (SMD = −0.23, 95% CI: −0.39 to −0.07) (Figure 3(b)). The certainty of evidence for this outcome is reported in Table 2.

##### 3.6.2.2. Subgroup Analysis

Subgroup analyses suggested that mixed and web‐based DAs were associated with a reduction in conflict (SMDs of −0.19 and −0.29, respectively), whereas the effect for brochure‐based DAs was uncertain (Figure 4(a) (B)). DAs targeting a specific cancer type showed a reduction in conflict (SMD = −0.26, 95% CI: −0.44 to −0.07), while those for mixed cancer types did not (Figure 4(b) (B)). Effects were observed in both Asian and Western regions (Figure 4(c)).

#### 3.6.3. Decision Satisfaction

Nine studies assessed decision satisfaction. The pooled analysis found no meaningful difference between groups (SMD = 0.03, 95% CI: −0.42–0.48) (Figure 3(c)). The wide CI is compatible with both a small benefit and a small harm from DAs, with the certainty of evidence for this outcome reported in Table 2.

The summary of the meta‐analysis on decision knowledge, decision conflict, and decision satisfaction is presented in Supporting Table S3.

## 4. Discussion

This systematic review and meta‐analysis encompassed 30 RCTs comprising a total of 4303 participants diagnosed with various types of cancer. We found that compared with the control group, DAs enhanced decision knowledge and reduced decision conflict among patients with cancer but did not improve their decision satisfaction.

### 4.1. The Quality of Evidence and Methodology

The quality of evidence ranged from low to moderate, primarily because of high heterogeneity and moderate methodological quality among the studies. Based on the modified Cochrane risk of bias assessment tool, 30% (nine studies) of the included studies showed a low risk of bias, 50% (15 studies) raised some concerns, and 20% (six studies) indicated a high risk of bias. Due to the nature of DAs, it is difficult to implement blinding for patients or researchers during the research. Additionally, most studies have longer intervention durations and include patients with advanced cancer, which may also be a reason for the missing data. For publication bias, the trim‐and‐fill method results in line with the original findings, suggesting robustness. Due to significant heterogeneity and potential biases in certain studies, the findings should be regarded with caution. Future research should concentrate on large‐scale, higher‐quality RCTs to gain a better understanding of how DAs affect the decision‐related outcomes of patients with cancer.

### 4.2. Principal Findings

#### 4.2.1. Decision Knowledge

Based on low‐certainty evidence, this meta‐analysis suggests that DAs may improve the decision knowledge of patients with cancer, in contrast to the conclusions drawn by earlier researchers [65]. Since the former included only six studies on decision knowledge, the limited amount of literature may be the reason for the discrepancy. DAs enable patients to respond to inquiries using common text, vivid images, and even videos to help them comprehend, which not only eases the doctor’s workload but also allows patients to fully prepare for SDM [66]. In addition, the data and options provided by DAs are grounded in high‐quality evidence that outlines the benefits, risks, and potential outcomes [67]. Thus, patients with cancer can use reliable sources to fully understand the knowledge related to their disease and improve their engagement and confidence in treatment.

The findings from subgroup analysis stratified by delivery methods indicated that DAs may improve decision knowledge among patients with cancer, though the effect varied by delivery methods. Mixed delivery methods demonstrated more consistent benefits and lower heterogeneity, suggesting their reliability in diverse settings. In contrast, web‐based delivery methods showed extreme variability, possibly due to differences in user engagement or design complexity. The high overall heterogeneity underscores the need for standardized interventions and subgroup analysis by patients’ characteristics. The subgroup analysis based on whether DAs were designed specifically for a single cancer population revealed significant differences in effectiveness. DAs tailored to a single cancer type demonstrated higher effect variability but included studies with notably strong effects. In contrast, nonspecific DAs showed more moderate heterogeneity and generally small effects. This suggests that population‐specific DAs may better address unique knowledge gaps or cultural needs, though their success likely depends on contextual factors like disease stage or intervention intensity.

To maximize the impact of DAs, future development should prioritize mixed delivery methods (e.g., written materials paired with clinician guidance), which demonstrated consistent benefits across studies [68].

#### 4.2.2. Decision Conflict

This meta‐analysis found that DAs effectively led to a reduction in decision conflict among patients with cancer, aligning with the findings of prior research [69]. DAs can decrease their conflict by offering information about options, benefits, and risks and employing tactics like presenting outcomes in a comprehensive manner, which includes detailing the physical, emotional, and social impacts, in order to enhance the understanding of patients’ personal value. Moreover, some interventions guide or coach patients with the help of medical staff, which can help them feel more supported in SDM [70, 71].

While the overall analysis suggested that DAs reduced conflict, the effect estimate for brochure‐based DAs was imprecise, with the confidence interval encompassing both a potential small reduction and a small increase in conflict. This high degree of uncertainty precludes a definitive conclusion about the effect of brochure‐based DAs alone. In contrast, mixed‐method approaches combining informational materials with interpersonal support consistently demonstrated small‐to‐moderate conflict reduction with lower heterogeneity. Future development should focus on optimizing brochure content for specific decision situations [72]. Moreover, the analysis comparing whether DAs were designed specifically for a single cancer population revealed that decision conflict decreased when the DAs targeted individual cancer types. This may be because the elements of decision conflict are variable and subjective, and the aim of individual cancer types can exhaustively consider patients’ specific reasons. For instance, some cancer patients experience decision conflict due to their precarious economic status, while others face conflict stemming from personal values [73, 74]. Our subgroup analysis revealed consistent efficacy of DAs in reducing decision conflict across both Asian and Western countries. This indicates that fundamental elements of DAs, including the provision of structured information and the clarification of values, may surpass cultural limitations in meeting the universal needs of patients in the context of medical decision‐making [75]. Nevertheless, the increased heterogeneity noted in studies conducted in Western contexts necessitates careful consideration; variations in healthcare systems across regions or inconsistencies in the implementation of DAs could potentially mask subtle cultural influences [76, 77].

Due to the different symptoms of cancer and the complex decision‐making dilemmas, nurses should develop DAs based on specific cancer populations. Moreover, these findings suggest that nurses should focus on optimizing core components, such as addressing information needs, clarifying values, and providing structured guidance [78, 79].

#### 4.2.3. Decision Satisfaction

The findings of this study proved that DAs are ineffective in enhancing decision satisfaction among patients with cancer, which is identified with the findings of previous studies [80]. Decision satisfaction is a highly personal reaction that can be influenced by various factors [81]. When cancer is diagnosed, patients often experience intense and intricate emotional responses, and feelings of anxiety and fear can impact their SDM [82]. These complex psychological issues cannot be addressed solely through DAs. To enhance cancer patients’ decision satisfaction, nurses can use social support systems such as psychological counselors and family members, which provide not only dependable informal but also emotional support [81, 84].

In our previous literature search, we found that DAs are commonly used for breast and prostate cancer survivors. Therefore, emotional support for these two kinds of patients can focus on the participation of spouses to jointly cope with decision dilemmas [85, 86]. Although subgroup analysis could not be performed, we suspect that the high heterogeneity is attributed to the various measurements utilized in the studies that were included.

### 4.3. Limitations

Potential limitations must also be recognized: (1) the search was restricted to English and Chinese articles, potentially introducing publication bias and impacting result reliability. (2) Differences in participant demographics, study characteristics, and the use of varied assessment tools likely contributed to heterogeneity. The limited number of included studies impedes further exploration of the sources of heterogeneity.

### 4.4. Strengths

This study offers both methodological rigor and clinically actionable insights, making it a valuable contribution to the field. We adhered to Cochrane guidelines by employing a random‐effects model to account for heterogeneity, conducted predefined subgroup analyses to explore regional variations, and followed PRISMA 2020 standards to ensure transparency. Beyond statistical robustness, our findings provide practical guidance for oncology nursing—confirming DAs’ effects in reducing conflict while highlighting the need to tailor interventions based on accessibility, health literacy, and cultural context. By integrating rigorous evidence synthesis with real‐world applicability, this study not only advances research methodology but also informs clinical strategies for SDM in cancer care [87].

### 4.5. Implications for Practice

The findings of this meta‐analysis provide strategies for integrating DAs into clinical oncology practice. Nurses should prioritize mixed delivery methods, which demonstrated consistent improvements in decision knowledge and conflict reduction. Additionally, DAs must be tailored to specific cancer populations (e.g., breast or prostate cancer) to address knowledge gaps and value‐based decision dilemmas, while DAs may not enhance satisfaction, leveraging psychological counseling and family involvement to address emotional barriers.

## 5. Conclusion

The results indicated that DAs could improve decision knowledge and decrease decision conflict. Delivery methods and whether DAs were designed specifically for a single cancer population had an impact on the efficacy of reducing decision conflict. Hence, its development should be prioritized, and it should be integrated into a part of routine practice; nurses should strengthen their communication skills to actively participate in the process; design DAs targeting individual cancer types, educational levels, and health literacy; utilize the advantages of various delivery methods; determine the cause of patients’ decision conflict; clarify their values; emphasize the influence of regional culture norms; and involve social support systems.

## Author Contributions

Yang Chen: conceptualization, methodology, software, data curation, validation, formal analysis, writing original draft preparation, and writing–review and editing.

Chuanmei Zhu: conceptualization, validation, and formal analysis.

Qianwen Yan: conceptualization, methodology, software, data curation, validation, formal analysis, writing original draft preparation, and writing–review and editing.

Linna Li: methodology, data curation, and writing–review and editing.

Juejin Li: conceptualization, validation, and formal analysis.

Xiaolin Hu: conceptualization, project administration, funding acquisition, and writing–review and editing.

## Funding

The study was supported by the National Natural Science Foundation of China (82473452 and 82172842), the China Medical Board (Grant# 22‐482), the Ministry of Education University‐Industry Collaborative Education Program (230720523707281), the Chengdu Eastern New Area Municipal Administration Committee, Bureau of Culture and Tourism Program (00402053A29YN), the Sichuan University Graduate Students Education Teaching Reform Research Program (GSSCU2023090 and GSSCU2023095), and the Chengdu Eastern New Area Technology Innovation Research and Development Program (2024‐DBXQ‐KJYF009).

## Conflicts of Interest

The authors declare no conflicts of interest.

## Supporting Information

Additional supporting information can be found online in the Supporting Information section.

## Supporting information


**Supporting Information** Supporting Table S1. Search strategies of databases and search engine. Supporting Figure S1. Risk‐of‐bias summary of the included studies. Supporting Table S2. Complete detailed characteristics of the 30 randomized controlled trials included in this review. Supporting Table S3. Summary of meta‐analysis on decision knowledge, decision conflict, and decision satisfaction. Supporting Figure S2. Results of Egger’s test and trim‐and‐fill methods. Supporting Figure S3. Sensitivity analysis for decision knowledge, decision conflict, and decision satisfaction.

## Data Availability

The datasets generated or analyzed during this study are available from the corresponding author on reasonable request.
