# Supplementary material for: Effects of Decision Aids on Decision Knowledge, Conflict, and Satisfaction Among Patients With Cancer: A Systematic Review and Meta‐Analysis
Source: J Nurs Manag. 2026 May 14;2026:6436400. doi: 10.1155/jonm/6436400 (PMC13176620; doi:10.1155/jonm/6436400)
Supplement: Supplementary file 1 — Supporting Information Supporting Table S1. Search strategies of databases and search engine. Supporting Figure S1. Risk‐of‐bias summary of the included studies. Supporting Table S2. Complete detailed characteristics of the 30 randomized controlled trials included in this review. Supporting Table S3. Summary of meta‐analysis on decision knowledge, decision conflict, and decision satisfaction. Supporting Figure S2. Results of Egger’s test and trim‐and‐fill methods. Supporting Figure S3. Sensitivity analysis for decision knowledge, decision conflict, and decision satisfaction. [file JONM-2026-6436400-s001.docx]

**Supplemental Materials**

**Supplementary Table S1.** Search strategies of Databases and Search engine.

**Supplementary Figure S1.** Risk-of-bias summary of the included studies.

**Supplementary Table S2.** Complete Detailed Characteristics of the 30 Randomized Controlled Trials.

**Supplementary Table S3.** Summary of meta-analysis on decision knowledge, decision conflict and decision satisfaction.

**Supplementary Figure S2.** Results of Egger’s test and trim-and-fill methods.

**Supplementary Figure S3.** Sensitivity analysis for decision knowledge, decision conflict and decision satisfaction.

**Supplementary Table S1.** Search strategies of Databases and Search engine

| **1. PubMed（1908-30, November, 2025）** | | |
| --- | --- | --- |
| #1 | Search: Neoplasms[MeSH Terms] | 4,105,641 |
| #2 | Search: Carcinoma[MeSH Terms] | 771,141 |
| #3 | Search: (((((((((((((neoplasm[Title/Abstract]) OR (malignant neoplasms[Title/Abstract])) OR (malignant[Title/Abstract])) OR (malignancy[Title/Abstract])) OR (malignancies[Title/Abstract])) OR (tumor[Title/Abstract])) OR (tumors[Title/Abstract])) OR (tumour[Title/Abstract])) OR (tumours[Title/Abstract])) OR (cancer[Title/Abstract])) OR (cancers[Title/Abstract])) OR (onco*[Title/Abstract])) OR (carcinoma[Title/Abstract])) OR (adenocarcinoma[Title/Abstract]) | 4,412,042 |
| #4 | #1 OR #2 OR #3 | 5,553,790 |
| #5 | Search: (((((((((((((((((((Technique, Decision Support[Title/Abstract]) OR (Techniques, Decision Support[Title/Abstract])) OR (Decision Support Technics[Title/Abstract])) OR (Decision Support Technic[Title/Abstract])) OR (Technic, Decision Support[Title/Abstract])) OR (Technics, Decision Support[Title/Abstract])) OR (Models, Decision Support[Title/Abstract])) OR (Decision Support Model[Title/Abstract])) OR (Decision Support Models[Title/Abstract])) OR (Model, Decision Support[Title/Abstract])) OR (Decision Modeling[Title/Abstract])) OR (Modeling, Decision[Title/Abstract])) OR (Decision Aids[Title/Abstract])) OR (Aid, Decision[Title/Abstract])) OR (Aids, Decision[Title/Abstract])) OR (Decision Aid[Title/Abstract])) OR (Decision Analysis[Title/Abstract])) OR (Analyses, Decision[Title/Abstract])) OR (Decision Analyses[Title/Abstract])) OR (Analysis, Decision[Title/Abstract]) | 31,248 |
| #6 | #4 AND #5 | 5,432 |
| **2. Embase（1966-30, November, 2025）** | | |
| #1 | 'neoplasm'/exp | 6,647,486 |
| #2 | 'carcinoma'/exp | 1,698,270 |
| #3 | neoplasm:ti,ab,kw OR 'malignant neoplasms':ti,ab,kw OR malignant:ti,ab,kw OR malignancy:ti,ab,kw OR malignancies:ti,ab,kw OR tumors:ti,ab,kw OR tumour:ti,ab,kw OR cancer:ti,ab,kw OR cancers:ti,ab,kw OR onco:ti,ab,kw OR carcinoma:ti,ab,kw OR adenocarcinoma:ti,ab,kw | 5,382,494 |
| #4 | #1 OR #2 OR #3 | 7,509,622 |
| #5 | 'technique, decision support':ti,ab,kw OR 'techniques, decision support':ti,ab,kw OR 'decision support technics':ti,ab,kw OR 'decision support technic':ti,ab,kw OR 'technic, decision support':ti,ab,kw OR 'technics, decision support':ti,ab,kw OR 'models, decision support':ti,ab,kw OR 'decision support model':ti,ab,kw OR 'decision support models':ti,ab,kw OR 'model, decision support':ti,ab,kw OR 'decision modeling':ti,ab,kw OR 'modeling, decision':ti,ab,kw OR 'decision aids':ti,ab,kw OR 'aid, decision':ti,ab,kw OR 'aids, decision':ti,ab,kw OR 'decision aid':ti,ab,kw OR 'decision analysis':ti,ab,kw OR 'analyses, decision':ti,ab,kw OR 'decision analyses':ti,ab,kw OR 'analysis, decision':ti,ab,kw | 19,301 |
| #6 | #4 AND #5 | 4,851 |
| **3. Web of Science Core Collection (1950–30, November, 2025)** | | |
| #1 | TI=(neoplasms) | 45,407 |
| #2 | TI=(carcinoma) | 547,975 |
| #3 | TI=(neoplasm OR malignant neoplasms OR malignant OR malignancy OR malignancies OR tumour OR tumors OR tumour OR cancer OR cancers OR onco OR carcinoma OR adenocarcinoma) | 3,312,563 |
| #4 | #1 OR #2 OR #3 | 3,312,563 |
| #5 | TI=(Technique, Decision Support OR Techniques, Decision Support OR Decision Support Technics OR Decision Support Technic OR Technic, Decision Support OR Technics, Decision Support OR Models, Decision Support OR Decision Support Model OR Decision Support Models OR Model, Decision Support OR Decision Modeling OR Modeling, Decision OR Decision Aids OR Aid, Decision OR Aids, Decision OR Decision Aid OR Decision Analysis OR Analyses, Decision OR Decision Analyses OR Analysis, Decision) | 49,207 |
| #6 | #4 AND #5 | 2,276 |
| **4. CENTRAL (1993–30, November, 2025)** | | |
| #1 | MeSH descriptor: [Neoplasms] explode all trees | 127,284 |
| #2 | MeSH descriptor: [Carcinoma] explode all trees | 20,476 |
| #3 | (neoplasm OR malignant neoplasms OR malignant OR malignancy OR malignancies OR tumour OR tumors OR tumour OR cancer OR cancers OR onco OR carcinoma OR adenocarcinoma):ti,ab,kw | 281,097 |
| #4 | #1 OR #2 OR #3 | 301,243 |
| #5 | (Technique, Decision Support OR Techniques, Decision Support OR Decision Support Technics OR Decision Support Technic OR Technic, Decision Support OR Technics, Decision Support OR Models, Decision Support OR Decision Support Model OR Decision Support Models OR Model, Decision Support OR Decision Modeling OR Modeling, Decision OR Decision Aids OR Aid, Decision OR Aids, Decision OR Decision Aid OR Decision Analysis OR Analyses, Decision OR Decision Analyses OR Analysis, Decision):ti,ab,kw | 20,583 |
| #6 | #4 AND #5 | 5,158 |
| **5. PsycINFO (1946–30, November, 2025)** | | |
| #1 | XB (cancer* OR tumor* OR tumour* OR onco* OR metastat* OR teratoma* OR neoplas* OR carcinoma* OR malignan* ) | 103,045 |
| #2 | XB (Technique, Decision Support OR Techniques, Decision Support OR Decision Support Technics OR Decision Support Technic OR Technic, Decision Support OR Technics, Decision Support OR Models, Decision Support OR Decision Support Model OR Decision Support Models OR Model, Decision Support OR Decision Modeling OR Modeling, Decision OR Decision Aids OR Aid, Decision OR Aids, Decision OR Decision Aid OR Decision Analysis OR Analyses, Decision OR Decision Analyses OR Analysis, Decision) | 85,416 |
| #3 | #1 AND #2 | 3,091 |
| **6. CINAHL (1937–30, November, 2025)** | | |
| #1 | TI ( neoplasm OR malignant neoplasms OR malignant OR malignancy OR malignancies OR tumor OR tumors OR tumour OR tumours OR cancer OR cancers OR onco* OR carcinoma OR adenocarcinoma ) OR AB ( neoplasm OR malignant neoplasms OR malignant OR malignancy OR malignancies OR tumor OR tumors OR tumour OR tumours OR cancer OR cancers OR onco* OR carcinoma OR adenocarcinoma ) | 7,523,764 |
| #2 | TI Technique, Decision Support OR Techniques, Decision Support OR Decision Support Technics OR Decision Support Technic OR Technic, Decision Support OR Technics, Decision Support OR Models, Decision Support OR Decision Support Model OR Decision Support Models OR Model, Decision Support OR Decision Modeling OR Modeling, Decision OR Decision Aids OR Aid, Decision OR Aids, Decision OR Decision Aid OR Decision Analysis OR Analyses, Decision OR Decision Analyses OR Analysis, Decision | 258,052 |
| #3 | #1 AND #2 | 3,684 |
| **7. CBMdisc (2008–30, November, 2025)** | | |
| #1 | "癌症"[标题:智能] OR "癌"[标题:智能] OR "肿瘤"[标题:智能] OR "瘤"[标题:智能] OR "恶性肿瘤"[标题:智能] OR "良性肿瘤"[标题:智能] | 3, 957, 720 |
| #2 | ("决策支持系统"[标题:智能] OR "临床决策支持"[标题:智能] OR "临床决策支持系统"[标题:智能]) OR ("决策支持技术"[标题:智能] OR "决策支持工艺"[标题:智能] OR "决策辅助"[标题:智能] OR "决策分析"[标题:智能] OR "决策模型"[标题:智能] OR "临床预测规则"[标题:智能]) | 8842 |
| #3 | (("决策支持系统"[标题:智能] OR "临床决策支持"[标题:智能] OR "临床决策支持系统"[标题:智能]) OR ("决策支持技术"[标题:智能] OR "决策支持工艺"[标题:智能] OR "决策辅助"[标题:智能] OR "决策分析"[标题:智能] OR "决策模型"[标题:智能] OR "临床预测规则"[标题:智能])) AND ("癌症"[标题:智能] OR "癌"[标题:智能] OR "肿瘤"[标题:智能] OR "瘤"[标题:智能] OR "恶性肿瘤"[标题:智能] OR "良性肿瘤"[标题:智能]) | 927 |
| **8. CNKI (1999–30, November, 2025)** | | |
| #1 | TKA='癌症' OR TKA='癌' OR TKA='肿瘤' OR TKA='瘤' OR TKA='良性肿瘤' OR TKA='恶性肿瘤' | 2,007,657 |
| #2 | TKA='决策辅助' OR TKA='决策辅助系统' OR TKA='决策辅助工具' OR TKA='决策辅助功能' OR TKA='决策辅助手段' OR TKA='决策辅助模型' OR TKA='决策辅助方法' OR TKA='决策辅助研究' OR TKA='决策辅助制度' OR TKA='决策辅助者' OR TKA='决策辅助标准' | 2,493 |
| #3 | TKA='决策支持' OR TKA='决策支持系统' OR TKA='决策支持技术' OR TKA='决策支持服务' OR TKA='决策支持信息' OR TKA='决策支持功能' OR TKA='决策支持平台' OR TKA='决策支持工具' | 57,512 |
| #4 | TKA='决策支持' OR TKA='决策支持系统' OR TKA='决策支持技术' OR TKA='决策支持服务' OR TKA='决策支持信息' OR TKA='决策支持功能' OR TKA='决策支持平台' OR TKA='决策支持工具' AND TKA='决策辅助' OR TKA='决策辅助系统' OR TKA='决策辅助工具' OR TKA='决策辅助功能' OR TKA='决策辅助手段' OR TKA='决策辅助模型' OR TKA='决策辅助方法' OR TKA='决策辅助研究' OR TKA='决策辅助制度' OR TKA='决策辅助者' OR TKA='决策辅助标准' | 58,187 |
| #5 | (TKA='决策支持' OR TKA='决策支持系统' OR TKA='决策支持技术' OR TKA='决策支持服务' OR TKA='决策支持信息' OR TKA='决策支持功能' OR TKA='决策支持平台' OR TKA='决策支持工具' AND TKA='决策辅助' OR TKA='决策辅助系统' OR TKA='决策辅助工具' OR TKA='决策辅助功能' OR TKA='决策辅助手段' OR TKA='决策辅助模型' OR TKA='决策辅助方法' OR TKA='决策辅助研究' OR TKA='决策辅助制度' OR TKA='决策辅助者' OR TKA='决策辅助标准)AND(TKA='决策支持' OR TKA='决策支持系统' OR TKA='决策支持技术' OR TKA='决策支持服务' OR TKA='决策支持信息' OR TKA='决策支持功能" OR TKA='决策支持平台' OR TKA='决策支持工具' AND TKA='决策辅助' OR TKA='决策辅助系统' OR TKA='決策辅助工具' OR TKA='決策辅助功能' OR TKA='决策辅助手段' OR TKA='决策辅助模型' OR TKA='决策辅助方法' OR TKA='决策辅助研究' OR TKA='决策辅助制度'OR TKA='决策辅助者' OR TKA='决策辅助标准)AND(TKA="癌症' OR TKA='癮' OR TKA='肿瘤' OR TKA='"瘤' OR TKA='良性肿瘤' OR TKA='恶性肿瘤”) | 382 |
| **9. WanFang (1998–30, November, 2025)** | | |
| #1 | 题名或关键词:(癌症) or 题名或关键词:(癌) or 题名或关键词:(肿瘤) or 题名或关键词:(瘤) or 题名或关键词:(良心肿瘤) or 题名或关键词:(恶性肿瘤) | 2,793,533 |
| #2 | 题名或关键词:(决策辅助) or 题名或关键词:(决策辅助系统) or 题名或关键词:(决策辅助工具) or 题名或关键词:(决策辅助功能) or 题名或关键词:(决策辅助手段) or 题名或关键词:(决策辅助模型) AND 题名或关键词:(决策辅助方法) or 题名或关键词:(决策辅助研究) or 题名或关键词:(决策辅助制度) or 题名或关键词:(决策辅助者) or 题名或关键词:(决策辅助标准) AND 题名或关键词:(决策支持) or 题名或关键词:(决策支持系统) or 题名或关键词:(决策支持技术) or 题名或关键词:(决策支持服务) or 题名或关键词:(决策支持信息) or 题名或关键词:(决策支持功能) | 41,231 |
| #3 | #1 AND #2 | 386 |
| **10. VIP (2000–30, November, 2025)** | | |
| #1 | ((((((((题名或关键词=癌症 OR 题名或关键词=癌)) OR 题名或关键词=肿瘤)) OR 题名或关键词=瘤)) OR 题名或关键词=良性肿瘤)) | 1,155,359 |
| #2 | ((((((((((((((((题名或关键词=决策辅助 OR 题名或关键词=决策辅助系统) OR 题名或关键词=决策辅助工具) OR 题名或关键词=决策辅助功能) OR 题名或关键词=决策辅助手段) OR 题名或关键词=决策辅助模型) OR 题名或关键词=决策辅助方法) OR 题名或关键词=决策辅助研究) OR 题名或关键词=决策辅助制度) OR 题名或关键词=决策辅助者) OR 题名或关键词=决策辅助标准) OR 题名或关键词=决策支持) OR 题名或关键词=决策支持系统) OR 题名或关键词=决策支持技术) OR 题名或关键词=决策支持服务) OR 题名或关键词=决策支持信息) OR 题名或关键词=决策支持功能) | 18,073 |
| #3 | ((((((题名或关键词=癌症 OR 题名或关键词=癌) OR 题名或关键词=肿瘤) OR 题名或关键词=瘤) OR 题名或关键词=良性肿瘤) OR 题名或关键词=恶性肿瘤) AND ((((((((((((((((题名或关键词=决策辅助 OR 题名或关键词=决策辅助系统) OR 题名或关键词=决策辅助工具) OR 题名或关键词=决策辅助功能) OR 题名或关键词=决策辅助手段) OR 题名或关键词=决策辅助模型) OR 题名或关键词=决策辅助方法) OR 题名或关键词=决策辅助研究) OR 题名或关键词=决策辅助制度) OR 题名或关键词=决策辅助者) OR 题名或关键词=决策辅助标准) OR 题名或关键词=决策支持) OR 题名或关键词=决策支持系统) OR 题名或关键词=决策支持技术) OR 题名或关键词=决策支持服务) OR 题名或关键词=决策支持信息) OR 题名或关键词=决策支持功能)) | 120 |

**Supplementary Figure S1.** Risk-of-bias summary of the included studies.

(A) Studies with intention-to-treat analysis


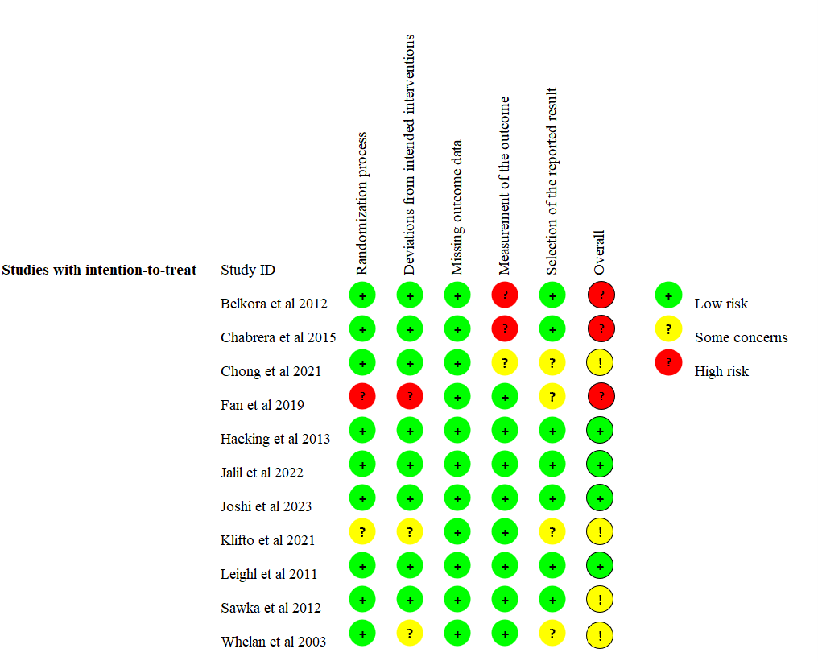


(B) Studies with per-protocol analysis


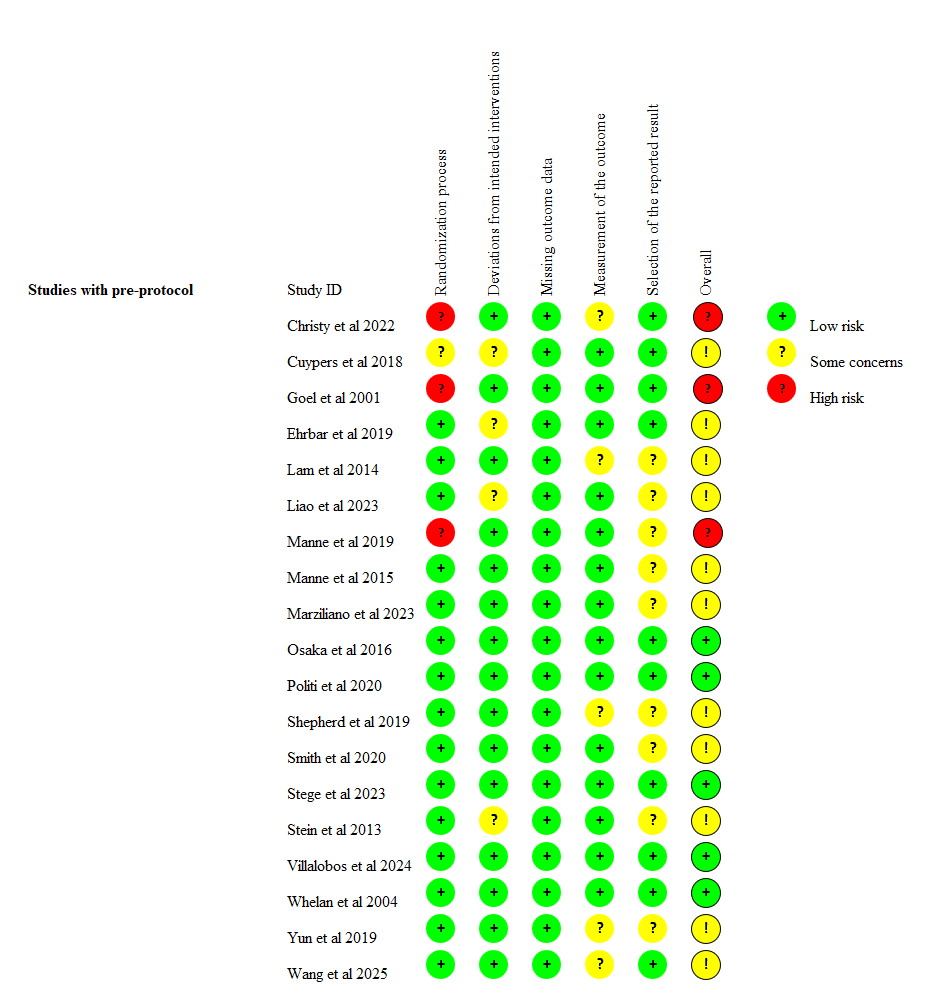


**Supplementary Table S2.** Complete Detailed Characteristics of the 30 Randomized Controlled Trials.

| **First author**  **Year, country** | **Study design and data analysis methods** | **Name (if had) & brief introduction** | **Duration** | **Control** | **Decision related Outcomes**  **(Measures)** | **Assessment** |
| --- | --- | --- | --- | --- | --- | --- |
| Belkora et al., 2012  USA [40] | Two-arm RCT  Intention-to-Treat | Deliver consultation planning (a question-listing intervention) by telephone | 6 months | UC | Satisfaction (The client satisfaction) | Post intervention |
| Chabrera et al., 2015  Spain [41] | Two-arm RCT  Intention-to-Treat | A printed booklet structured in 2 parts | 3 months | UC | Knowledge (A 5-item inquiry)  Conflict (DCS)  Satisfaction (SWD) | Post intervention |
| Chong et al., 2021  Singapore [42] | Two-arm RCT  Intention-to-Treat | Structured guidance in decision making on oral CAM use and promoting discussions with healthcare professionals | 1 month | UC | Knowledge (A brief self-made questionnaire)  Conflict (DCS) | 1 months after intervention |
| Christy et al., 2022  USA [26] | Two-arm RCT  Per-protocol | "CHOICESDA" is a web-based, plain language decision aid | 2 weeks | AC  (NCI CCT Website) | Knowledge (Eleven items survey)  Conflict (A 4-item screening test) | Post intervention |
| Cuypers et al., 2018  Netherlands [43] | Two-arm RCT  Per-protocol | Focus on facilitating SDM patients received access to the online DA in addition to usual information and counseling | 3 weeks | UC | Knowledge (An estimation of the perceived knowledge level)  Conflict (The Dutch version of DCS)  Satisfaction (SCIP) | Post intervention |
| Fan et al., 2019  China [44] | Two-arm RCT  Intention-to-Treat | Evidence-based cancer treatment manual, with patient-friendly PPT visuals | 1 week | UC | Satisfaction (PSMDS) | Two days before the patient is discharged |
| Goel et al., 2001  USA [45] | Two-arm RCT  Per-protocol | The decision aid workbook content is geared to a grade 8 reading level. The audiotape recorded in a comforting female voice | 6 months | UC | Conflict (DCS) | 6 months after intervention |
| Ehrbar et al., 2019  Switzerland [46] | Two-arm RCT  Per-protocol | The online DA was developed by an interdisciplinary team of specialists in reproductive medicine, gynecologist, oncologists and psychologists | 12 months | UC | Conflict (DCS) | 12 months after counselling |
| Hacking et al., 2013  UK [47] | Two-arm RCT  Intention-to-Treat | A "navigator" guiding the patient in creating a personal question list for a consultation and providing a CD and typed summary of the consultation | 6 months | UC | Conflict (DCS) | 6 months after the consultation |
| Jalil et al., 2022  Malaysia [48] | Two-arm RCT  Intention-to-Treat | A printed comprehensive treatment booklet designed for patients recently diagnosed with localized prostate cancer | 1 month | UC | Knowledge (A validated knowledge questionnaire)  Conflict (DCS) | 1 month after the consultation |
| Joshi et al., 2023  India [49] | Three-arm RCT  Intention-to-Treat | "Navya PPT": a self-administered, online, adaptive, conjoint analysis-based decision aid and patient preference assessment tool | NA | UC | Conflict (DCS) | Post intervention |
| Klifto et al., 2021  USA [50] | Two-arm RCT  Intention-to-Treat | A decision aid brochure, originally developed by the Stanford University Medical Center | 2 weeks | UC | Conflict (DCS) | 2 weeks after consultation |
| Lam et al., 2014  China [51] | Two-arm RCT  Per-protocol | The standard-information booklet contained information on diagnosis, treatment, and management of breast cancer in general terms | 10 months | UC | Knowledge (NR) Conflict (NR) | 10 months after intervention |
| Leighl et al., 2011  Canada [52] | Two-arm RCT  Intention-to-Treat | The DA was developed as a booklet with accompanying narration on an audiotape or compact disc for patients to take home | 4 weeks | UC | Conflict (DCS)  Satisfaction (SWD) | 2 weeks after treatment decision |
| Liao et al., 2023  China [27] | Two-arm RCT  Per-protocol | A nurse-led decision support intervention consists of two main parts: decision aids and decision guidance. | 2-30 days | UC | Knowledge (HCC treatment-related knowledge scale)  Conflict (The Chinese version of DCS)  Satisfaction (SWD) | Post intervention |
| Manne et al., 2019  USA [24] | Two-arm RCT  Per-protocol | "B-sure", an online decision support aid to facilitate informed decisions regarding CPM | 4 weeks | UC | Knowledge (A 10-item multiple-choice scale) | 2-4 weeks after surgery |
| Manne et al., 2015  USA [53] | Two-arm RCT  Per-protocol | "BRAID" is a menu-driven program organized into 10 modules. | 2 weeks | UC | Knowledge (A 19-item knowledge survey)  Conflict (DCS)  Satisfaction (A seven-item measure) | 2 weeks after intervention |
| Marziliano et al., 2023  USA [54] | Two-arm RCT  Per-protocol | "Healing Choices" is a multimedia software program that provides information and decision-making support for women with early-stage breast cancer. | 2 months | UC | Conflict (DCS) | 2 months after intervention |
| Osaka et al., 2016  Japan [55] | Three-arm RCT  Per-protocol | This booklet contains information about methods for the diagnosis of breast cancer and explanations of diagnosis and treatment procedures | 12 months | UC | Conflict (DCS) | 1 month after surgery |
| Politi et al., 2020  USA [56] | Two-arm RCT  Per-protocol | "I Can PIC" : Improving Cancer Patients' Insurance Choices | 6 months | AC  (A health insurance worksheet) | Knowledge (The Henry J. Kaiser Foundation’s health insurance quiz) | 3-6 months after intervention |
| Sawka et al., 2012  Canada [57] | Two-arm RCT  Intention-to-Treat | The DA describes the rationale, possible risks and benefits, and the medical evidence uncertainty relating to the choice | 12 months | UC | Knowledge (A medical questionnaire)  Conflict (DCS) | A mean of 6.3 months after initial random assignment |
| Shepherd et al., 2019  UK [58] | Two-arm RCT  Per-protocol | "CPRS" combines three evidence-based practices: coached question listing followed by audio recording and then summarizing of the consultation | 3 months | UC | Conflict (DCS) | After third medical consultation |
| Smith et al., 2020  USA [59] | Two-arm RCT  Per-protocol | The online Four Conversations program | 4 weeks | UC | Conflict (DCS) | 4 weeks after intervention |
| Stege et al., 2023  Netherlands [60] | Two-arm RCT  Per-protocol | An online decision aid aims to prepare patients for consultation with a plastic surgeon | 12 months | UC | Conflict (DCS) | 3 months after surgery |
| Stein et al., 2013  Australia [61] | Two-arm RCT  Per-protocol | A pamphlet called "Living with Advanced Cancer" | 3 months | UC | Knowledge (The knowledge questionnaire) | Post intervention |
| Villalobos et al., 2024  German [62] | Two-arm RCT  Per-protocol | The decision aid is a brochure which queries information needs and decision style (preference in participation) and identifies the patient’s personal values | NR | UC | Conflict (DCS) | Post intervention |
| Whelan et al., 2004  Canada [28] | Two-arm RCT  Per-protocol | The Decision Board is a visual aid that presents written and graphical information from clinical trials to patients regarding their treatment options | 12 months | UC | Conflict (DCS)  Satisfaction (The effective decision-making subscale) | 12 months after surgery |
| Whelan et al., 2003  Canada [63] | Two-arm RCT  Intention-to-Treat | The Decision Board is a visual aid that presents written and graphical information from clinical trials to patients regarding their treatment options | 12 months | UC | Knowledge (A 25-item questionnaire) | 1 week after consultation |
| Yun et al., 2019  Korea [64] | Two-arm RCT  Per-protocol | Received and viewed a 20-minute decision-support video on a notebook computer and a companion 43-page book developed entitled Advanced Care Planning | 7 weeks | AC  (Video + Book about controlling cancer pain) | Knowledge (Self-made Questionnaire)  Conflict (DCS) | Seven weeks after intervention |
| Wang et al., 2025  China [65] | Two-arm RCT  Per-protocol | A web-based decision aid included multiple modules such as health information, and decision evaluation, support, and assessment | 1 months | AC  (Paper-based DAs) | Conflict (DCS)  Satisfaction (PSMDS | T1: 1 day before surgery |

^a^: at baseline；ACSCAN: American Cancer Society’s Cancer Action Network；BRAID: Web-based Breast Reconstruction Decision Support Aid；C: Control group；CPRS: Consultation Planning Recording Summarizing；CPM: Contralateral prophylactic mastectomy; CSQ: The client satisfaction questionnaire；DA: Decision aid；DCS: Decision Conflict Scale；E: Experimental group；HCC: Hepatocellular Carcinoma；Navya-PPT: Navya-Patient；Preference Tool；NR: Not reported；NCI CCT Website: the National Cancer Institute Cancer Clinical Trials website；PSMDS: the Participation Satisfaction in Medical Decision-making Scale；SDM: Shared Decision Making；UC: Usual care/Standard care；AC: Attention control；SWD: The Satisfaction With Decision Scale；SCIP: The Satisfaction with Cancer Information Profile

**Supplementary Table S3.** Summary of meta-analysis on decision knowledge, decision conflict and decision satisfaction.

| **Outcomes** | **Subgroup** | | | **No. of**  **Studies** | **No. of Participants**  **(experimental/control)** | **Pooled effect size**  **[95% *CI*]** | ***p* Value for**  **pooled result** | **I^2^ (%)** | ***p* Value for**  **heterogeneity** |
| --- | --- | --- | --- | --- | --- | --- | --- | --- | --- |
| **Decision knowledge** |  | | | 15 | 1130/991 | [0.49, 1.33] | 0.00 | 94.93 | 0.00 |
|  | Delivery methods | | |  |  |  |  |  |  |
|  | | Brochure | | 6 | 453/428 | [0.07, 0.87] | 0.00 | 87.66 | - |
|  | | Mixed | | 3 | 198/208 | [0.34, 0.74] | 0.70 | 0.00 | - |
|  | | Web-based | | 6 | 479/355 | [1.22, 3.64] | 0.00 | 97.82 | - |
|  | Whether designed specifically for targeted cancer population | | |  |  |  |  |  |  |
|  | | No | | 6 | 485/510 | [0.27, 0.79] | 0.00 | 74.78 | - |
|  | | Yes | | 9 | 645/481 | [0.78, 2.35] | 0.0001 | 96.87 | - |
| **Decision conflict** |  | | | 24 | 1799/1701 | [-0.39, -0.07] | 0.00 | 80.16 | - |
|  | Delivery methods | | |  |  |  |  |  |  |
|  | | Brochure | | 9 | 587/607 | [-0.53, 0.18] | 0.00 | 88.80 | - |
|  | | Mixed | | 6 | 528/525 | [-0.37, -0.02] | 0.09 | 47.51 | - |
|  | | Web-based | | 9 | 684/569 | [-0.54, -0.04] | 0.00 | 76.14 | - |
|  | Whether designed specifically for targeted cancer population | | |  |  |  |  |  |  |
|  | | | No | 4 | 259/272 | [-0.31, 0.17] | 0.16 | 41.69 | - |
|  | | | Yes | 20 | 1540/1429 | [-0.44, -0.07] | 0.00 | 82.49 | - |
|  | Conducted regions | | |  |  |  |  |  |  |
|  | Asian countries | | | 8 | 552/496 | [-0.37, -0.04] | 0.10 | 41.27 | - |
|  | Western countries | | | 16 | 1247/1205 | [-0.47, -0.02] | 0.00 | 85.55 | - |
| **Decision satisfaction** |  | | | 9 | 709/572 | [-0.42, 0.48] | 0.90 | 93.25 | 0.00 |

**Supplementary Figure S2.** Results of Egger’s test and trim-and-fill methods

Outcome 1: Decision conflict

**
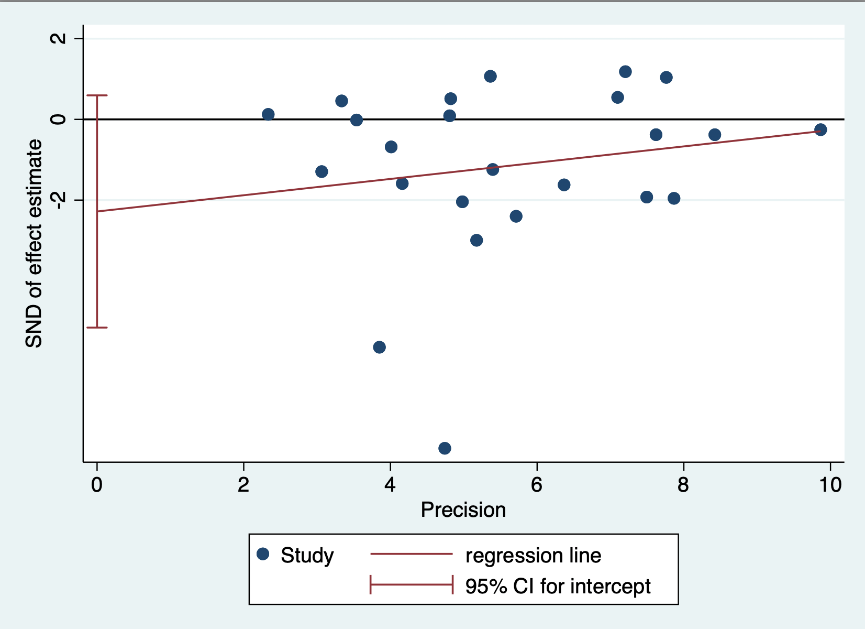
**

**
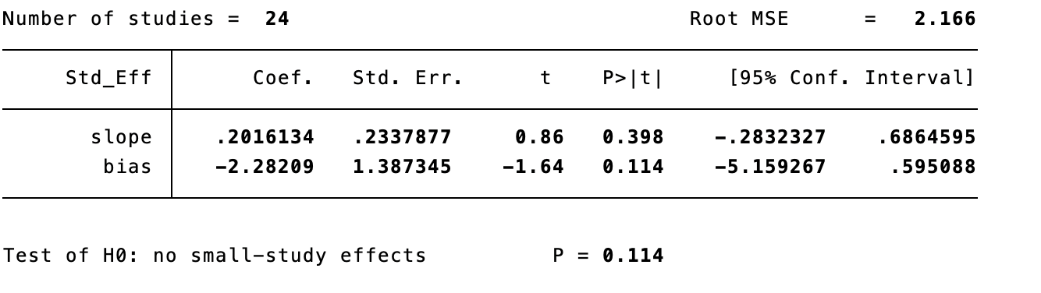
**

Outcome 2: Decision knowledge

**
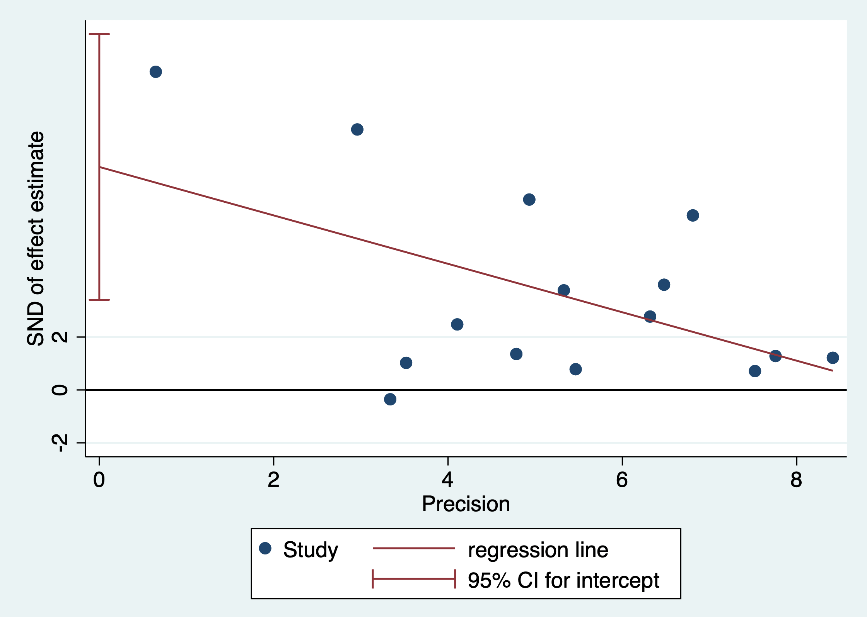

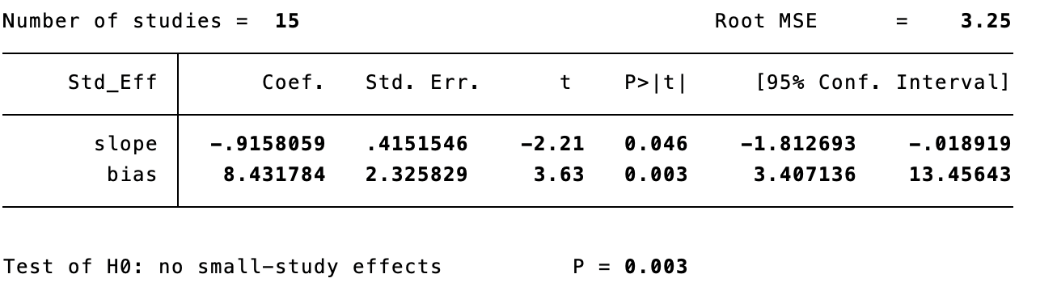
**

**Supplementary Figure S3.** Sensitivity analysis for decision knowledge, decision conflict and decision satisfaction.

| **Outcomes** | **Egger’s test (*P*)** | **Meta-analysis results**  **SMD [95% CI]** | **Trim-and-fill methods****  **SMD [95% CI]** |
| --- | --- | --- | --- |
| Decision knowledge | 0.003* | 0.91 [0.49, 1.33] | 0.907 [0.486, 1.327] |
| Decision conflict | 0.114 | -0.23 [-0.39, -0.07] | - |
| Decision satisfaction | - | 0.03 [-0.42, 0.48] | - |

***** *P*<0.05

****** The stochastic effect model is used for trimming and filling analysis. If the deviation is corrected, the SMD and 95% CI estimates are indicated.

SMD: Standardized Mean Difference

Outcome 1: Decision knowledge

**
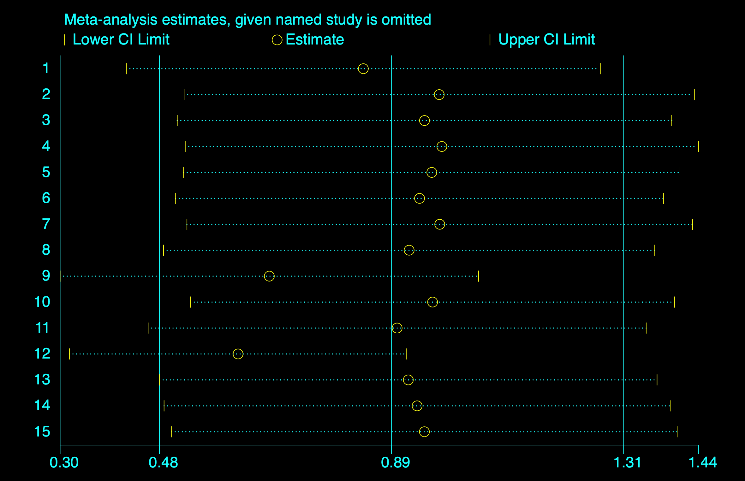
**

Outcome 2: Decision conflict

**
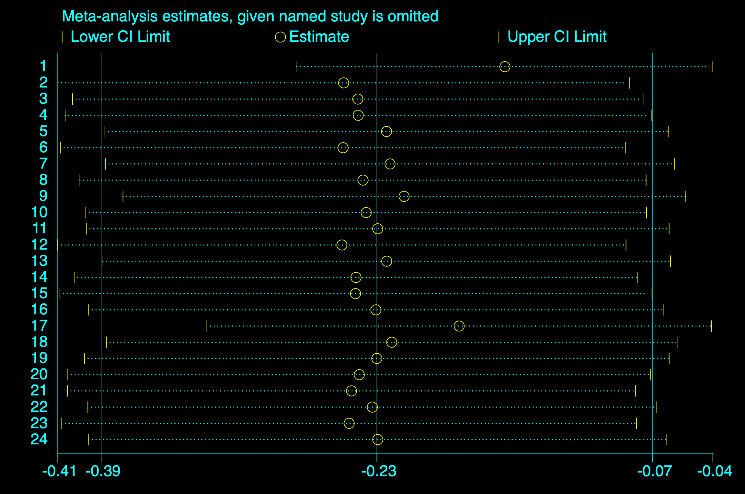
**

Outcome 3: Decision satisfaction

**
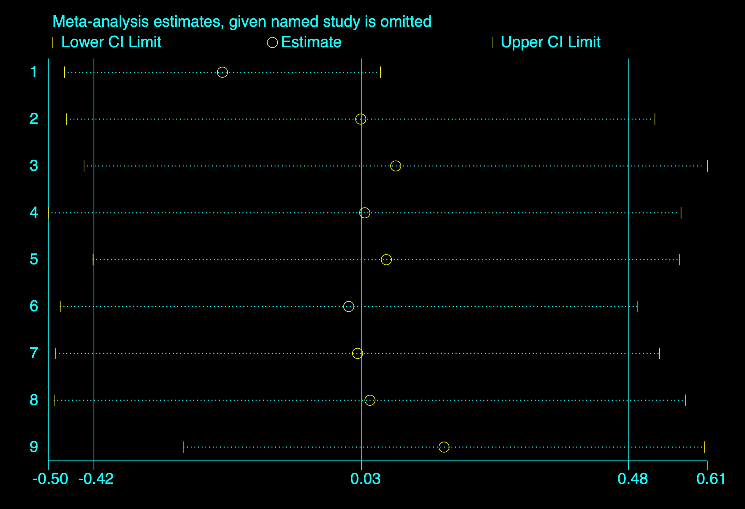
**
